# Occipital Nerve Stimulation for Chronic Migraine—A Systematic Review and Meta-Analysis

**DOI:** 10.1371/journal.pone.0116786

**Published:** 2015-03-20

**Authors:** Yen-Fu Chen, George Bramley, Gemma Unwin, Dalvina Hanu-Cernat, Janine Dretzke, David Moore, Sue Bayliss, Carole Cummins, Richard Lilford

**Affiliations:** 1 School of Health and Population Sciences, University of Birmingham, Birmingham, United Kingdom; 2 School of Psychology, University of Birmingham, Birmingham, United Kingdom; 3 University Hospitals Birmingham NHS Foundation Trust, Birmingham, United Kingdom; 4 Division of Health Sciences, University of Warwick, Coventry, United Kingdom; University of Würzburg, GERMANY

## Abstract

**Background:**

Chronic migraine is a debilitating headache disorder that has significant impact on quality of life. Stimulation of peripheral nerves is increasingly being used to treat chronic refractory pain including headache disorders. This systematic review examines the effectiveness and adverse effects of occipital nerve stimulation (ONS) for chronic migraine.

**Methods:**

Databases, including the Cochrane Library, MEDLINE, EMBASE, CINAHL and clinical trial registers were searched to September 2014. Randomized controlled trials (RCTs), other controlled and uncontrolled observational studies and case series (n≥ 10) were eligible. RCTs were assessed using the Cochrane risk of bias tool. Meta-analysis was carried out using a random-effects model. Findings are presented in summary tables and forest plots.

**Results:**

Five RCTs (total n=402) and seven case series (total n=115) met the inclusion criteria. Pooled results from three multicenter RCTs show that ONS was associated with a mean reduction of 2.59 days (95% CI 0.91 to 4.27, I2=0%) of prolonged, moderate to severe headache per month at 3 months compared with a sham control. Results for other outcomes generally favour ONS over sham controls but quantitative analysis was hampered by incomplete publication and reporting of trial data. Lead migration and infections are common and often require revision surgery. Open-label follow-up of RCTs and case series suggest long-term effectiveness can be maintained in some patients but evidence is limited.

**Conclusions:**

While the effectiveness of ONS compared to sham control has been shown in multiple RCTs, the average effect size is modest and may be exaggerated by bias as achieving effective blinding remains a methodological challenge. Further measures to reduce the risk of adverse events and revision surgery are needed.

**Systematic Review Registration:**

this systematic review is an update and expanded work of part of a broader review registered with PROSPERO. Registration No. CRD42012002633.

## Introduction

Migraine is a debilitating primary headache disorder that has been ranked as the eighth leading cause in terms of years lived with disability globally [1]. While most people with migraine have ‘episodic migraine’ (defined as having <15 days of headache each month), each year about 2.5% of the sufferers have their headache ‘transformed’ into chronic migraine [2], which has even greater impact on the level of disability, social functioning and use of healthcare resources compared to episodic migraine [3].

Chronic migraine is defined as “headache occurring on 15 or more days per month for more than 3 months, which has the features of migraine headache on at least 8 days per month” in the latest International Classification of Headache Disorders, 3rd edition beta version (ICHD-III beta) [4]. An alternative term ‘transformed migraine’ which emphasises the nature of headache that is developed from episodic migraine with increasing headache frequency but decreasing severity of migraine features has also been used in the literature [5]. The diagnostic criteria and definitions for chronic migraine have been evolving over time with different requirements regarding the number and feature of headache days and varied exclusion criteria in relation to medication overuse headache [6]. Medication overuse headache refers to chronic headache that is developed as a consequence of regular overuse of acute or symptomatic headache medication such as paracetamol, anti-inflammatory drugs, ergotamine, triptans and opioids [4]. It occurs commonly among migraine sufferers and is a risk factor contributing to the development from episodic to chronic migraine. Medication withdrawal may revert chronic migraine back to episodic migraine [3]. In ICHD-II, medication overuse needs to be absent for a diagnosis of chronic migraine to be made [7], but a concomitant diagnosis of chronic migraine and medication overuse headache is allowed in ICHD-III beta [4] and is indeed used in common practice. As controversies over the optimal definition of chronic migraine are yet to be resolved, we use the term ‘chronic migraine’ in the rest of this paper for consistency, but use it to include chronic or transformed migraine in its various manifestations.

Treatments for chronic migraine aim to reduce the frequency of migraine attacks and associated disability [3]. Many drugs for the prophylaxis of episodic migraine have been used (often off-label) for chronic migraine [3]. Only topiramate and botulinum toxin type A are supported by evidence from large randomised trials of patients with chronic migraine [3, 6, 8]. Despite the advances in the management of headache disorders, many patients with chronic migraine remain refractory to current treatments—a recent study in a tertiary headache centre in Spain showed that 15 of 20 patients with chronic migraine fulfilled the criteria for refractory chronic migraine [9]. Novel treatments backed by good evidence are therefore in much need.

Electrical stimulation of peripheral nerves has been used to treat various painful conditions including headache disorders[10]. Occipital nerve stimulation (ONS) is one of the invasive techniques that is gaining popularity for treating chronic migraine[11]. Continuous stimulation of occipital nerves is achieved by the delivery of electrical impulses through cylindrical or paddle electrodes (leads) implanted subcutaneously. The procedure is usually done in two stages, involving an initial trial of stimulation of a few days to a couple weeks which, if successful, is followed by a permanent implant [12].

ONS may have a plausible biological basis [13] and has been shown to affect blood flow in brain structures thought to be important in the patho-physiology of migraine [14]. Early case series in patients with chronic migraine were summarized in a systematic review and showed promising results [15]. These were followed by randomized controlled trials (RCTs) reporting mixed findings [16–19]. As these RCTs were not included the previous review and further case series have been published, this systematic review aims to critically appraise this growing body of evidence and provide an overview to inform clinical and policy decisions.

## Methods

This systematic review was initially conducted as part of a broad review of evidence on the use of invasive peripheral nerve stimulation for treating chronic refractory pain in support of the development of Interventional Procedures Guidance by the UK National Institute for Health and Care Excellence (NICE) [20]. The protocol for the broad review was registered with PROSPERO (Registration No. CRD42012002633). This paper reports an updated review focusing on ONS for chronic migraine.

### Search strategy and selection criteria

The following electronic databases were searched (inception to September 2014):

- The Cochrane Library (Wiley)
- MEDLINE and MEDLINE In Process (Ovid), EMBASE (Ovid) and CINAHL (EBSCO)
- The ZETOC (Mimas) database, and Conference Proceedings Citation Index (ISI Web of Knowledge).
- Current Controlled Trials metaRegister, NIHR Clinical Research Network Portfolio, WHO International Clinical Trials Registry Platform (ICTRP), and ClinicalTrials.gov for ongoing studies.

Searches were conducted using index terms and key words relating to peripheral nerve stimulation, chronic pain, headache disorders, migraine and occipital nerve stimulation. No language restriction was applied. A sample search strategy can be found in Appendix A in S1 File. Relevant websites including the Medicines and Healthcare Products Regulatory Agency (MHRA) and Food and Drug Administration (FDA) were searched and reference lists of included studies were scanned. Studies highlighted during NICE’s public consultation of relevant Interventional Procedures Guidance were also examined.

At least two reviewers independently carried out the study selection. A study was included if it:

- Recruited patients with chronic migraine, defined according to the International Classification of Headache Disorders, 2^nd^ edition (ICHD-II)[7] or its subsequent modification [21]. Studies that adopted diagnostic criteria for transformed migraine suggested by Silberstein and colleagues prior to ICHD-II were also included [5].
- Investigated the effect of stimulation of occipital nerves or areas innervated by them.
- Was an RCT or non-randomized controlled study, or an uncontrolled case series with at least 10 patients.
- Reported results for chronic migraine separately where various types of headache disorders were included.

We included studies in migraine patients where all participants were refractory to multiple prophylactic treatments but the proportion of patients with chronic migraine (as oppose to episodic migraine) was not clearly reported. Non-English studies or those published only as conference abstracts were excluded, with the exception of RCTs which were included irrespective of publication status. Studies that focused on the combined use of ONS with other forms of nerve stimulation were excluded.

### Data extraction and assessment of risk of bias

Details on study design, trial participants, techniques of nerve stimulation, funding sources, effectiveness findings, and adverse events were extracted. For effectiveness, outcome measures recommended by the International Headache Society’s guidelines were used [22].

The Cochrane Collaboration’s Risk of Bias Tool was used to appraise RCTs [23]. Information regarding the effectiveness of blinding and patients’ expectation of treatment was also noted. Data extraction and assessment of risk of bias was carried out by one reviewer and quality assured by another. Any discrepancies in study selection, extraction and assessment were resolved through discussions. Case series were used to provide supplementary evidence on stimulation trials, adverse events and longer-term effectiveness. No separate risk of bias assessment was carried out for them. The overall quality of evidence for selected key outcomes was rated according to the GRADE framework [24].

### Data synthesis

Characteristics of included studies and the results of risk of bias assessment were tabulated. Assessment of effectiveness focused on evidence from RCTs. Where suitable data was available (see Appendix B in S1 File), meta-analyses of risk ratios (for binary outcomes) and mean differences (for continuous outcomes) were carried out in Review Manager 5.2 using a random-effects model. Statistical heterogeneity between studies was evaluated by visual inspection of forest plots and the I^2^ statistic. No statistical assessment of potential publication bias was carried out due to the relatively small number of included RCTs. Authors and sponsors of partially reported RCTs were contacted for unpublished data, although none were supplied.

As the majority of the adverse events were related to surgical procedures and implanted devices (rather than to the stimulation *per se*), data concerning adverse events from the active stimulation and sham control arms of the RCTs were combined (unless otherwise specified) and presented alongside additional data from case series. The proportion of patients who experienced specific adverse events were displayed in forest plots with 95% confidence interval calculated using the exact method [25]. Meta-analysis was not carried out for adverse events given the varied methods of data collection, event classification and length of follow-up between studies.

## Results

**Fig 1:**
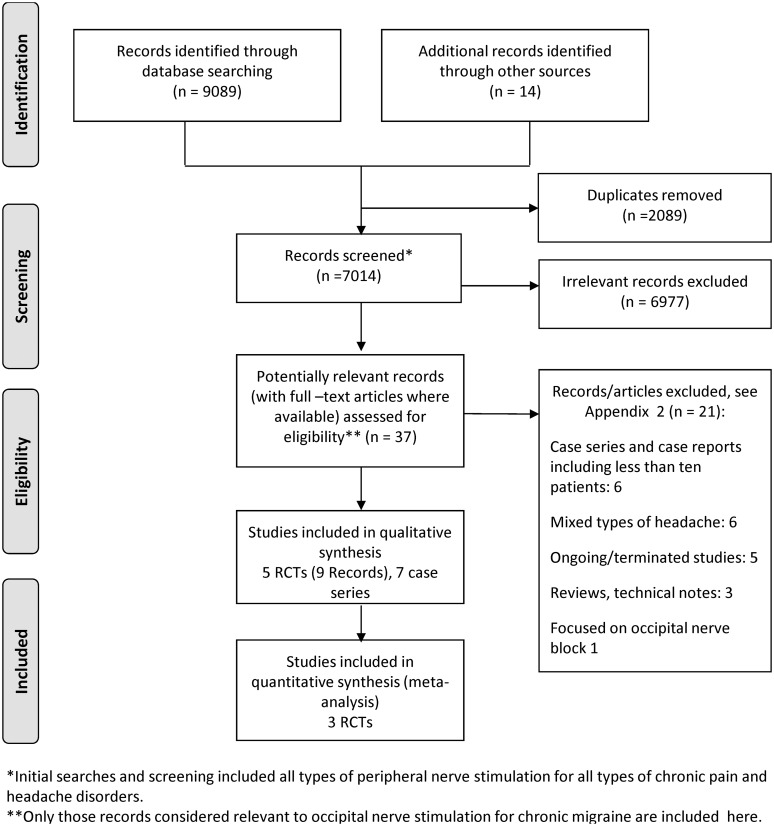
Flow diagram for study selection.

Five RCTs (reported in nine publications [17–19, 26–31]) and seven case series [32–38] met the inclusion criteria (Fig. 1). In addition, one unpublished [39] and one ongoing RCT [29] was identified. The unpublished RCT (the UK PRISM study) was terminated early (with only eight patients enrolled) based on interim data from another RCT [16] (PRISM study, included in this review) sponsored by the same manufacturer. A list of excluded studies with reasons for exclusion can be found in Appendix C in S1 File.

### Characteristics of included studies

Of the five included RCTs (Table 1), three were industry-sponsored, multicentre, parallel-group trials (PRISM study—published only as a conference abstract [16], ONSTIM study [17], and Silberstein et al. 2012 [19]) and two were single-centre crossover trials (Serra and Marchioretto 2012 [18], Slotty et al. 2014 [31]). The sample size ranged from 67 to 157 in the multicentre RCTs and was eight and 30 for each of the two single centre RCTs respectively.

**Table 1:** Characteristics of included randomized controlled trials.

Study, location & sample size (n randomized)	Centre, design & treatment arms (n analysed)*	Diagnostic criteria	Treatment history	Patients with medication overuse	Trial stimulation and/or nerve block required	Follow-up	Sponsorship & comments
Lipton et al. 2009 (PRISM study),[16] USA, n = 140	Multicentre, parallel-group, ONS (n = 63) vs. sham (n = 62)	ICHD-2 (migraine with or without aura, and/or chronic migraine)	Refractory to ≥ 2 acute and ≥2 prophylactic medications	Included (pre-specified subgroup)	Trial stimulation done but success was not an inclusion criteria	Double-blind 12 weeks, uncontrolled open label 1 year	Industry sponsored; published only as a conference abstract
Saper et al. 2011 (ONSTIM study),[17] USA, Canada & UK, n = 67	Multicentre, parallel-group, ONS (n = 28) vs. sham (n = 16) vs. medication management (n = 17)	ICHD-2 (chronic migraine)	Refractory to ≥ 2 classes of prophylactic medications	Excluded	Successful temporary nerve block (≥50% reduction in pain) required	Single-blind 12 weeks, uncontrolled open-label 3 years	Industry sponsored; also included an non-randomized ancillary arm (n = 8), in which patients who did not respond to occipital nerve block received active ONS
Silberstein et al. 2012,[28, 30] USA, n = 157	Multicentre, parallel-group, ONS (n = 105) vs. sham (n = 52)	ICHD-2 (chronic migraine) with modification using the Silberstein-Lipton diagnostic criteria for transformed migraine	Refractory to ≥ 2 acute and ≥2 classes of prophylactic medications	Possibly included (through the criteria for transformed migraine)	Successful trial stimulation (≥50% reduction in pain or adequate paresthesia) required	Double-blind 12 weeks, uncontrolled open-label 1 year	Industry sponsored
Serra, Marchioretto, 2012,[18] Italy, n = 30	Single centre, crossover, ‘ONS ‘on’ vs ‘off’ (n = 29)	Chronic migraine or medication overuse headache	Refractory to ≥ 2 prophylactic medications	Included	Successful trial stimulation (≥50% in the number or severity of attacks) required	Controlled open-label 2 x 1 month (no washout period), uncontrolled open-label 1 year	Hospital-based, no external funding
Slotty et al. 2014, [31] Germany, n = 8	Single centre, crossover, Suprathreshold vs subthreshold vs no stimulation (n = 8)	IHS criteria for chronic migraine	Treated with ONS & reported >30% pain relief for ≥3 months, on stable medication	Not described	All patients already had good response to ONS—see ‘Treatment history’	Double-blind (except suprathreshold stimulation), 3 x 1 week (no washout period)	No external funding

*The numbers of patients actually included in the analyses by study authors

ICHD-2: International Classification of Headache Disorder 2nd edition, IHS: International Headache Society, ONS: occipital nerve stimulation.

All three multicentre RCTs included an initial blinded phase of 12 weeks, during which patients received either active or sham stimulation.[16, 17, 19] The blinded phase was followed by an open label phase of 1–3 years during which all participants received active stimulation (results not yet published). The ONSTIM study also included a third arm of medication management group [17], which could be regarded as an open-label control group given that the patients were already refractory to medication management when entering the study. The single-centre crossover RCT by Serra and Marchioretto was designed as an open-label study with ONS switched on in one group and off in another group for a month. The two groups were then crossed over for another month [18]. However, patients in the ‘off’ group could switch their stimulation on if they had ≥30% worsening in the number or severity of migraine attacks, and they did so after an average of just under five days. All patients had their ONS switched on after two months and continued to be followed up for ten further months. The other single-centre crossover RCT [31] compared suprathreshold stimulation (stimulation that was felt to be effective in reducing pain), subthreshold stimulation (stimulation with amplitude just below perception) and no stimulation. Patients received each of the stimulation options for one week in random order with no washout period between them. Patients and physicians were not aware of treatment allocation but suprathreshold stimulation cannot be effectively blinded. [31] The study included patients who had already had good response (>30% pain relief) to ONS for at least three months. It was set out to assess the significance of paresthesia and possible placebo effects of ONS rather than effectiveness and safety.

Stimulation trials were carried out in three RCTs [16, 18, 19]. Occipital nerve blocks and intraoperative testing were performed in the fourth [17]. Successful trial stimulation or nerve block was a criterion for permanent implantation and study enrolment in all studies except the PRISM study. Trial stimulation was unsuccessful in between 3% to 11% of the patients screened in the trials [17–19]. Patients in all studies were refractory to at least two prophylactic medications. Those who experienced medication overuse were excluded in the ONSTIM study [17]. Baseline migraine days per month were similar across the studies (between 20 to 23).

The seven case series included a total of 115 patients (Appendix D in S1 File) [32–36]. Duration of follow-up ranged from 1 to 79 months for individuals. Two of the case series included patients enrolled in RCTs described above [33, 34]. Stimulation parameters for all included studies are listed in Appendix E in S1 File.

### Assessment of risk of bias

The results of the risk of bias assessment for included RCTs are summarised in Table 2. The assessment for the PRISM study was hampered by the lack of full-text publication [16]. Of the other two multicentre RCTs, the risk of bias was judged to be high for the ONSTIM study for incomplete outcome data (15% dropout excluded from analysis in the ONS group compared to 6% in the sham group and 0% in the medication management group) and selective outcome reporting (data were not reported for several statistically non-significant results) [17]. The Silberstein et al. 2012 study was judged to be at low or unclear risk of bias [19], with the main uncertainty related to the effectiveness of blinding. Given that achieving paresthesia is considered a prerequisite for treatment effectiveness and a requirement before permanent implementation and trial enrolment in most studies, it is perceivable that a genuine sham control would be very difficult to attain. However, the success of blinding was not measured in any of the trials. The single-centre crossover RCT by Serra and Marchioretto was considered to be subject to high risk of bias in several domains due to lack of blinding, high level of contamination between groups and other issues related to crossover design [18]. Finally the other single-centre crossover trial by Slotty and colleagues [31] was judged to be of low risk for potential biases related to blinding for the comparison between subthreshold stimulation and no stimulation but of high risk of bias for comparisons against suprathreshold stimulation. The selection of patients (who had already had good response to ONS), short treatment duration and lack of washout periods rendered it to be judged as unsuitable for the purpose of assessing the effectiveness and safety of ONS for treating chronic migraine. Consequently, its findings are used only to inform discussion concerning placebo effects.

**Table 2:** Risk of bias assessment of included randomized controlled trials.

Bias domain	Source of bias	Lipton et al. 2009 (PRISM study) [16]	Saper et al. 2011 (ONSTIM study) [17]	Silberstein et al. 2012 [19]	Serra and Marchioretto, 2012 [18]	Slotty et al. 2014 [31]
Selection bias	Random sequence generation	Unclear risk	Low risk	Low risk	Unclear risk	Low risk
	Allocation concealment	Unclear risk	Low risk	Low risk	Unclear risk	Low risk
Performance bias	Blinding of participants	Unclear risk	Unclear risk (high risk for medication management group)	Unclear risk	High risk	Low risk (high risk for suprathreshold stimulation)
	Blinding of study personnel	Unclear risk	Unclear risk	Unclear risk	High risk	Low risk
Detection bias	Blinding of outcome assessment: patient reported outcomes	Unclear risk	Unclear risk (high risk for medication management group)	Unclear risk	High risk	Low risk (high risk for suprathreshold stimulation)
	Blinding of outcome assessment: investigator assessed outcomes	Low risk	Low risk	Low risk	High risk	Low risk
Attrition bias	Incomplete outcome data	Unclear risk	High risk	Low risk	Low risk	Low risk
Reporting bias	Selective reporting	Unclear risk	High risk	Unclear	Low risk	Low risk
Other bias	Any other important concerns about bias not covered in the other domains above	Based on conference abstract with very limited information; manufacturer-sponsored study	Manufacturer-sponsored study	Manufacturer-sponsored study	High risk (weakness related to crossover design—see below)	High risk (patients already had good treatment response; lack of washout—see below)
	Measurement of effectiveness of blinding and/or patients’ expectation of treatment effectiveness	Not done	Not done	Not done	Not done	Not done
Crossover design	Analysis of paired data	Not applicable	Not applicable	Not applicable	Not done	Yes
	Assessment of carryover effects and/or justification of washout period	Not applicable	Not applicable	Not applicable	Not done	Not done

### Effectiveness

**Fig 2:**
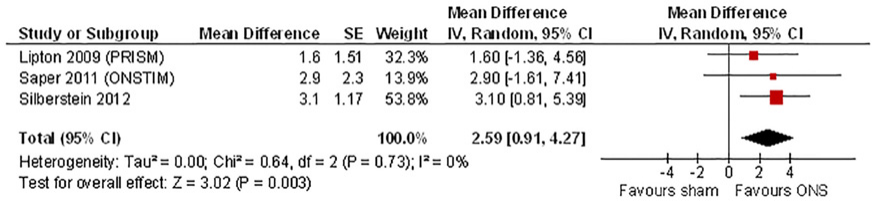
Results of meta-analysis of RCT data for ONS compared with sham stimulation: days with prolonged (≥4 hours) moderate or severe headache.

**Fig 3:**
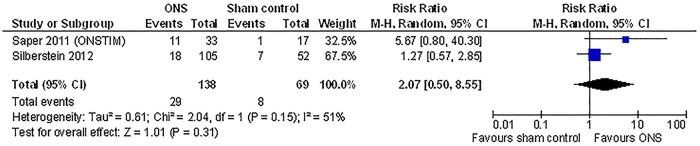
Results of meta-analysis of RCT data for ONS compared with sham stimulation: response rate.

Outcomes from RCTs were reported in different formats and were of various completeness (Appendix B in S1 File). Narrative and tabulated summaries are provided (see text below and Table 3). Meta-analyses were performed for two outcomes (Figs. 2 and 3).

**Table 3:** Additional short-term effectiveness results from randomized controlled trials.

Outcome measures	Saper et al. 2011 (ONSTIM study)(n = 67)	Silberstein et al. 2012 (n = 157)	Serra & Marchioretto, 2012 (n = 30)
**Headache (migraine) days**	**Mean reduction in headache days per month at 3 months**:	Not reported	**Median headache days per week at 1 month** (before crossover):
	ONS (n = 28): 6.7±10.0		ONS on: 2.1
	Sham (n = 16): 1.5±4.6 (p = 0.02)		ONS off: 6.3 (p<0.001)
	Medication management (n = 17): 1.0 ±4.2 (p = 0.008)		
Headache intensity*	**Mean reduction in overall pain intensity (0–10 scale) at 3 months**:	**Patient-reported percentage headache pain relief at 3 months**:	**Median headache severity (0–10 scale) at 1 month**:
	ONS: 1.5±1.6	ONS (n = 105): 42%	ONS on: 5
	Sham: 0.5±1.3 (p = 0.02)	Sham (n = 52): 17% (p<0.05)	ONS off: 7.5 (p<0.001)
	Medication management: 0.6 ±1.0 (p = 0.02)		
**MIDAS scores**	**Mean change in MIDAS average grade at 3 month**:	**Difference in mean reduction in MIDAS scores at 3 months**	**Median MIDAS total score (interquartile range)**, both groups combined** (n = 29):
	ONS: 0.4±0.8	ONS vs. sham, 44.1,	Baseline: 79 (30–135)
	Sham: not reported	95% CI 22.8 to 65.3	3 months: 19 (0–44)
	Medication management: 0.0 ±0.0 (p = 0.02)	(p = 0.001).	
**Utilization of acute medication**	**Mean reduction in acute medication use at 3 months**:	Not reported	**Median monthly doses of triptans**, both groups combined** (n = 22):
	ONS: 1.6±7.6		Baseline: 20; 3 months: 3
	Sham: not reported		**Median monthly doses of NSAIDs**,
	Medication management: -0.6 ±5.0 (p = 0.24)		both groups combined** (n = 16)
			Baseline: 25.5; 3 months: 3

MIDAS: Migraine Disability Assessment; NSAIDs: non-steriodal anti-inflammatory drugs; ONS: occipital nerve stimulation. Results from Lipton et al. 2009 (PRISM study, n = 140) were not published. P values shown are in comparison with the ONS group.

*Additional results (one-week treatment, n = 8) from Slotty et al. are described in Appendix S6 in S1 File.

**Comparative data not reported.

#### Days with prolonged (≥4 hours) moderate or severe headache

This outcome was reported in three multi-centre RCTs [16, 17, 19]. Patients in the trials had between 19–22 days with prolonged, moderate or severe headache per month at baseline. Those receiving sham stimulation had a reduction of 2–4 days per month at three months compared to baseline. Meta-analysis shows that ONS was associated with an additional mean reduction of 2.59 days per month (95% CI 0.91 to 4.27, I^2^ = 0%) compared with sham control. The results were consistent across studies (Fig. 2).

Lipton et al. stated in their conference abstract that in a pre-specified subgroup analysis for this outcome, a trend in favour of patients without medication overuse (ONS vs. sham, reduction of 5.9 vs. 2.6 migraine days/month) was observed compared with patients with medication overuse (ONS vs. sham, reduction of 5.0 vs. 4.8 migraine days/month) [16]. However, results for a formal test of interaction for the difference between subgroups were not presented.

#### Responder rate

The two studies that reported responder rates adopted a threshold of 50% improvement but defined the outcome differently (see Appendix B in S1 File) [17, 19]. The pooled result did not reach statistical significance and suggested a high level of heterogeneity between the studies (ONS vs. sham control at 3 months, relative risk 2.07, 95% CI 0.50 to 8.55, I^2^ = 51%; Fig. 3). Silberstein et al additionally conducted a continuous proportion responder analysis, which showed that the difference between groups became significant when the threshold of pain relief was reduced to 30% or lower (% responder 35% vs. 17% for ONS vs. sham control, p = 0.02 using 30% pain relief as the threshold) [19].

#### Other effectiveness outcomes

Results for the reduction in headache (migraine) days, headache intensity, Migraine Disability Assessment (MIDAS) scores and utilization of acute medications are summarized in Table 3. Additional data on quality of life, satisfaction of treatment and measures of physical and mental functions are described in Appendix F in S1 File. While the results are significantly better in the ONS group compared to the control group for the majority of outcomes, the reporting of results was incomplete and in some instances related to the lack of statistical significance of the findings [17]. The observed sizes of effects also varied widely for some of the outcomes. For example the difference in mean reduction in headache days between ONS and sham control was approximately 5 days *per month* in the ONSTIM study [17], but a much larger difference was reported in the open-label trial (median headache day *per week* 2.1 vs. 6.3 for ONS vs. control respectively) [18].

#### Long-term effectiveness

One the three multicentre RCTs reported findings from long-term follow-up beyond the initial blinded phase [30]. Additional long-term data (≥ one year) were available from the single centre RCT by Serra & Marchioretto [18] and six of the case series [32–34, 36–38]. As patients in the control groups of the RCTs also received ONS after the initial blinded phase, no long-term comparative data is available. The long-term effectiveness results are summarised in Appendix G in S1 File. Overall, more than 80% of patients continued to use ONS at 1 year across various studies, although the continuation rate seems to drop to around 50–60% in case series with longer follow-up of varied duration. The short-term effectiveness of ONS appears to be maintained in patients who stayed on the treatment.

### Adverse events

Results from both RCTs and case series are presented here. Data from intervention and sham control arms are combined within each RCT unless otherwise specified.

#### Serious adverse events

Serious adverse events occurred in between 1% (2/157) [19] to 6% (3/56) [17] of patients in multicentre RCTs at 3 months. Forty of the 209 adverse events (19%) recorded at 1 year in the trial by Silberstein et al. were classified as serious adverse events, of which 58% (23/40) were not considered as treatment-related and 35% (14/40) were related to lack of efficacy or return of symptoms. Up to 20% (2/10) [33] of patients experienced serious adverse events in smaller case series of varied duration of follow-up. Six out of nine patients (67%) in the terminated UK PRISM trial experienced serious adverse events [39]. The majority of treatment-related serious adverse events reported in these studies (except those related to lack of efficacy) were associated with infection, lead migration, post-operative symptoms and psychiatric complications (causality unclear). Further details are provided in Appendix F in S1 File.

#### Lead migration/dislodgement

**Fig 4:**
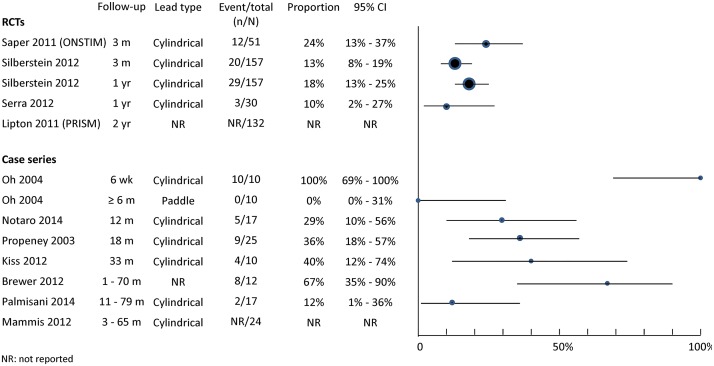
Adverse effects associated with implantation and/or use of occipital nerve stimulation: lead migrations.

Lead migration/dislodgement was common (Fig. 4). As expected higher rates were seen in case series with longer follow-up. One case series [35] suggests that using paddle-type leads rather than cylindrical leads can reduce the occurrence of lead migration. Measures were instigated during the ONSTIM trial to reduce lead migration [17], including the use of circular coils when placing the lead extension to create strain-relief loops, and choosing the abdomen in preference of the buttock as the implant location for the pulse generator. However, the impact of these measures was not formally assessed.

#### Infection

**Fig 5:**
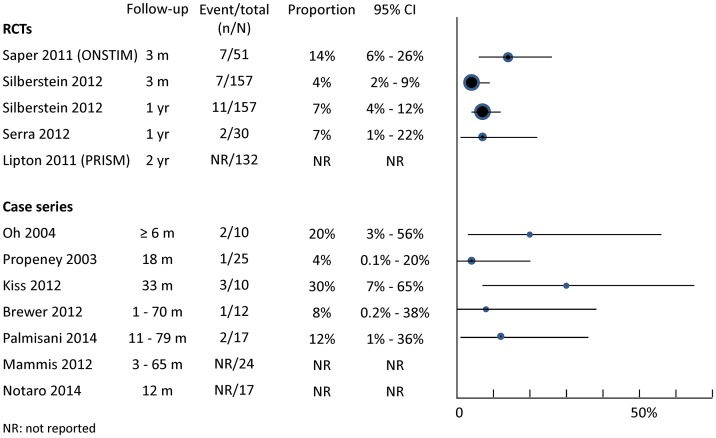
Adverse effects associated with implantation and/or use of occipital nerve stimulation: infections. Saper 2011—the number shown was infections at site for lead/extension tract. There were additionally four ‘complications at incision sites’.[17] Silberstein et al. 2012—there were additionally ‘wound site complications’ (four at 3 months;[28] five at 1 year[30]). Lipton et al. described infections being the most frequent device-related adverse events but did not report the numbers in their published abstract.[16] The three cases described by Kiss and colleagues were ‘inflammation at surgical sites’ (3/10, 30%) that were treated with intravenous and oral antibiotics.[33] They stated that ‘neither blood nor wound cultures identified bacterial growth’.

Reported infection rates range from 4% to 30% with varied length of follow-up (Fig. 5). The exact infection rates were difficult to ascertain in some studies due to the different ways in which infections were described and classified (see footnote for Fig. 5).

#### Other adverse events

Seventy-one percent (111/157) of patients experienced one or more adverse events over one year in the RCT by Silberstein and colleagues. Of a total of 209 adverse events recorded, 56 (27%) were hardware-related (e.g. battery failure, device malfunction or disconnection etc.), 82 (39%) were ‘biological’ events (e.g. infection, skin erosion, pain, numbness or swelling, allergic reaction, hematoma etc.), 45 (22%) were stimulation-related (e.g. unintended change in headache severity or stimulation, lack of efficacy, muscle spasms/cramping, nausea/vomiting etc.) and the remaining 26 (12%) were not considered treatment-related. Eighteen (9%) of the adverse events required hospitalization and 85 (41%) resulted in an additional surgery. Similar types of adverse events were reported in other RCTs and case series.

#### Long-term complications or potential nerve damage

Two trials reported that there was no evidence of adverse device effects leading to long-term complications or potential nerve damage (at three months [17] and one year [18] respectively). A case of subcutaneous tissue change at implant site and another case of reduction or loss of musculoskeletal control were reported in Silberstein et al [19].

## Discussion

This systematic review examines current evidence for using ONS for chronic migraine. Comprehensive literature search was performed, which identified five RCTs and seven case series that met the inclusion criteria. Findings from the RCTs suggest that at 3-month follow-up ONS reduces the number of days with prolonged moderate/severe headache by approximately 2.5 days per month compared to sham stimulation (which in itself brings about a reduction of 2–4 days per month compared to baseline). Responder analysis at 3 months using a threshold of 50% reduction in headache days and/or pain intensity favoured ONS but did not reach statistical significance in either individual trials or pooled results. ONS was shown to be more effective than sham control for other outcome measures, but results were often reported incompletely and in different formats, hindering the analysis of evidence across studies. Overall the level of evidence is considered moderate to low according to the GRADE framework [24] (see Appendix H in S1 File for Summary of Findings Table).

The short-term results indicate that the effect of ONS is, on average, modest among patients with chronic refractory migraine although the observed effects may still be clinically important given the refractory nature of the condition. Some individual patients experienced significant improvement that lasted for years in case series but the data is limited. On the other hand, adverse events associated with the devices and surgical procedures including lead migration and infections remain relatively common. These findings suggest that while ONS may be a valuable option when patients have exhausted other non-invasive treatments, further improvement in both efficacy and safety may be needed before it can firmly be established within the treatment pathway. Further large reductions in pain are desired by patients and associated with improvements in other outcomes including improved quality of life [40, 41]. To date the proportion of patients who achieved a response (albeit short of “no worse than mild pain”) in the larger trials has not been very high even at three months.

The definition of chronic migraine has evolved over time and this is reflected in the varied inclusion criteria for the RCTs included in our review. Whether the overall effectiveness of ONS can be improved by refining diagnostic and patient selection criteria remains to be seen. Limited evidence suggested that response to occipital nerve block may not be a useful predictor for response to ONS treatment[42]. While a subgroup analysis from the PRISM trial [16] and a small case series [43] suggested that ONS is more effective in patients without medication overuse, the observed effectiveness was similar between the ONSTIM trial (which excluded patients with medication overuse) and the other trials which allowed patients with medication overuse, including the crossover trial by Serra and Marchioretto in which 85% of the patients with chronic migraine also met the criteria for medication overuse [18].

A previous systematic review published in 2008 included only case series [15]. The identification in the current review of five completed and an ongoing RCT [29] and registry [44] is encouraging. In particular, three of the completed RCTs were industry-sponsored multicentre trials, which incorporated a sham control group with some attempts of blinding. The successful completion of these trials exemplifies that assessment of interventional procedures need not rely upon case series only. Despite this, the incomplete publication and reporting of results from ONS trials is an ongoing concern, the existence of which goes against the current movement of making all trial results available [45].

This systematic review has some limitations. Firstly, while the comprehensive search also attempted to retrieve unpublished data, none was made available and therefore the review findings are based upon published data only. Given the consistency in findings in the key outcome (reduction in prolonged, moderate/severe headache) reported in the three larger RCTs, the potential publication and reporting bias is likely to influence the estimation of effect sizes for other incompletely reported outcomes rather than change the direction of effect. Secondly, despite the existence of published guidelines for chronic migraine trials [22], the synthesis of evidence was hindered by the different ways in which outcomes were selected and reported in individual studies. Moreover, long-term data is limited. Apart from the one year results of the RCT by Silberstein and colleagues[30], evidence is available from predominantly single-centre case series, which could only provide imprecise estimations with uncertain generalizability.

An area of uncertainty for the RCT findings relates to how effective the blinding was in each of the trials, and how much this influenced the observed results. Patients with refractory migraine who are willing to try an invasive intervention are likely to have high expectations of the efficacy of the intervention [46], and there is some evidence that differential expectation due to presence or absence of blinding could impact upon observed clinical effects [47]. Apart from attempts to directly measure the success of blinding, future trials also need to pay attention to potential unblinding through patients’ sharing of experiences in social media as highlighted by Goadsby [48].

Slotty and colleagues compared suprathreshold stimulation, subthreshold stimulation (stimulation just below perception) and no stimulation in a small double-blind, crossover trial of ONS responders [31]. Although the issue of blinding remains for the suprathreshod stimulation, the observed differences in the reduction in pain intensity between each of the groups (see Appendix F in S1 File) suggests that while paresthesia contributes to the analgesia, the effect of ONS seems to go beyond merely a placebo effect induced by paresthesia.

In conclusion, currently evidence on the effectiveness and safety of ONS is still limited in quantity and remains inconclusive given the challenges in trial methodology and patient selection. Further development of ONS and other similar techniques and validation of their efficacy require both continuous accumulation of clinical evidence (for which multicentre, prospective registries may have an important role to play) and further studies on the pathophysiology of migraine and its responses to various forms of neuromodulation. In the interim, the use of ONS may be best guided by individual patient preference, affordability, treatment response and contribution to the accumulation of the evidence base through RCTs or prospective registries [49].

## Supporting Information

S1 File Appendices. (DOCX)

S1 Checklist PRISMA 2009 Checklist (DOCX)
