# Supplementary material for: Occipital Nerve Stimulation for Chronic Migraine—A Systematic Review and Meta-Analysis
Source: PLoS One. 2015 Mar 20;10(3):e0116786. doi: 10.1371/journal.pone.0116786 (PMC4368787; doi:10.1371/journal.pone.0116786)
Supplement: S1 File — (DOCX) [file pone.0116786.s001.docx]

**S1 File: Appendices**

Appendix A: Sample search strategy for MEDLINE

Database: Ovid MEDLINE(Ovid)

Search Strategy*

1 (stimulat$ adj peripheral nerve).mp.

2 peripheral nerve field.mp.

3 ((peripheral or percutaneous or subcutaneous or epicranial or epifacial or infraorbital or occipital or sacral or suboccipital or supraorbital or trigeminal or medial plantar) adj (nerve stimulat$ or neuromodulation or neurostimulat$)).mp.

4 ((occipital or sacral or suboccipital or supraorbital or trigeminal or percutaneous or subcutaneous or large fibre or subcutaneous target or conditioning electric$ or epifacial electric$ or sensory nerve or selective nerve root) adj stimulat$).mp

5 electroacupuncture.mp. or exp Electroacupuncture/

6 exp Electric Stimulation Therapy/

7 exp Peripheral Nervous System/

8 6 and 7

9 1 or 2 or 3 or 4 or 5 or 8

10 pain.mp. or exp Pain/

11 headache$.mp. or exp Headache/

12 migraine$.mp. or exp Migraine Disorders/

13 failed back surgery syndrome.mp. or exp Failed Back Surgery Syndrome/

14 FBSS.mp.

15 exp Complex Regional Pain Syndromes/ or complex regional pain.mp.

16 CRPS.mp.

17 causalgia.mp. or exp Causalgia/

18 reflex sympathetic dystrophy.mp. or exp Reflex Sympathetic Dystrophy/

19 angina.mp. or exp Angina Pectoris/

20 exp Neuralgia/ or neuralgia.mp.

21 sciatica.mp. or exp Sciatica/

22 neuropathy.mp.

23 hemicrania.mp.

24 SUNCT Syndrome/ or SUNCT.mp.

25 or/10-24

26 9 and 25

27 limit 26 to humans

*The search strategy was used in an initial broad search covering all types of pain conditions. The search was conducted in March 2012 and subsequently updated in December 2012. A further updated search was carried out in September 2014 using similar terms but focused only on migraine and headaches.

Appendix B: Outcomes measured and reported in the included RCTs

| **Outcomes** | **Lipton et al. 2009 (PRISM) [**[**1**](#_ENREF_1)**]** | **Saper et al. 2011 (ONSTIM) [**[**2**](#_ENREF_2)**]** | **Silberstein et al. 2012 [**[**3**](#_ENREF_3)**,** [**4**](#_ENREF_4)**]** | **Serra & Marchioretto 2012** **[**[**5**](#_ENREF_5)**]†** | **Slotty et al. 2014 [**[**6**](#_ENREF_6)**]** |
| --- | --- | --- | --- | --- | --- |
| Days with prolonged moderate or severe headache* | Yes | Yes (days per month and percentage reduction) | Yes | No | No |
| Headache days | Not reported | Yes (days per month and percentage reduction) | Not reported | Yes (days per week, median & IQR) | No |
| Headache intensity (pain intensity/ severity) | Not reported | Yes (pain intensity, 0-10 scale); also reported MIDAS headache pain score | Yes (VAS, but results not reported); presented patient- reported headache pain relief as categorical data and percentages | Yes (NRS-11, median & IQR) | Yes (VAS and McGill Pain Questionnaire) |
| Duration of headache | Not reported | Yes (change in hours of headache per day) | Not reported | Not reported | Not reported |
| Responder rate (≥30% or ≥50% reduction)* | Not reported | ≥50% reduction in number of headache days per month or ≥3 points reduction in pain intensity compared with baseline | ≥50% reduction in pain intensity with no increase in headache duration; also reported a continuous proportion responder analysis (i.e. % patients considered as responders at various threshold from 10%, 20%, 30%...to 100%) | Not reported | Not reported |
| Acute treatment utilization | Not reported | Yes (only reported significant results) | Not reported | Yes (results from two trial arms were lumped together) | Not reported |
| Conversion to episodic headache | Not reported | Not reported | Not reported | Not reported | Not reported |
| MIDAS | Not reported | Yes (changes in ‘MIDAS average grade’) | Yes (MIDAS score) | Yes (results from two trial arms were lumped together) | Not reported |
| Quality of life | Not reported | SF-36 (only reported significant results) | Yes (categorical rating of improved, stayed the same or deteriorated) | SF-36 (results from two trial arms were lumped together) | SF-36 |
| Satisfaction towards treatment | Not reported | Yes (only reported significant results) | Yes (yes or no) | Not reported | Not reported |
| Other outcomes | Not reported | Profile of Moods States, functional disability (only reported significant results) | Zung Pain and Distress Scale (at 52 weeks; results from two trial arms were lumped together) | Not reported | Not reported |

IQR – inter-quartile range; NRS – Numerical Rating Scale; SD – standard deviation; VAS – visual analogue scale.

*Sufficient data were reported in suitable formats and allowed meta-analyses to be performed for this outcome.

†Due to lack of blinding and a washout period, along with a high level of contamination (virtually all patients in the control group switched on their stimulators within two weeks due to worsening symptoms) in both phases (before and after cross-over) of this trial, only the comparative results from the first phase of this study are described in this review, and these data were not included in meta-analyses. Data from the uncontrolled phases (in which all patients received ONS) of the trial were presented alongside evidence from case series. Also the values for means and standard deviations were not reported for some of the outcomes.

Appendix C: List of excluded studies and reasons for exclusion

| **Study** | **Reason(s) for exclusion** |
| --- | --- |
| Abhinav K, Park ND, Prakash SK, Love-Jones S, Patel NK. Novel use of narrow paddle electrodes for occipital nerve stimulation--technical note. Neuromodulation. 2013; 16(6): 607-609. | Case series of five patients, only one of whom had chronic migraine. |
| Ducic I, Felder JM III, et al. A systematic review of peripheral nerve interventional treatments for chronic headaches. Annals of Plastic Surgery. 2014; 72(4): 439-445. | Systematic review covering various peripheral nerve interventional procedures and headache disorders – does not report results for ONS and chronic migraine patients separately. |
| Boindi D, Hammond J. Occipital nerve stimulation in the treatment of migraine (Verona). ClinicalTrials.gov 2013. Accessed 6 October 2014. Available from: http://clinicaltrials.gov/show/NCT01855672 | Study terminated due to business strategic decision; cannot verify the nature of the intervention (neuro-modulation stimulation E-box). |
| Falowski S, Wang D, Sabesan A, Sharan A, Falowski S, Wang D, et al. Occipital nerve stimulator systems: review of complications and surgical techniques. Neuromodulation. 2010; 13(2): 121-5. | Types of headache were not stated & results were not reported separately for migraine patients. |
| Fontaine D, Lanteri-Minet M. Efficacy evaluation of great occipital nerve electrical stimulation on rebound headache. ClinicalTrials.gov 2012. Accessed: 2 July 2012. Available from: <http://clinicaltrials.gov/ct2/show/NCT01184222> | Headache associated with withdrawal of medications in migraine patients with medication overuse associated with non-specific analgesics. |
| Franzini A, Messina G, Leone M, Broggi G. Occipital nerve stimulation (ONS). Surgical technique and prevention of late electrode migration. Acta Neurochirurgica. 2009; 151(7): 861-5. | Mixed type of headache – mainly cluster headache. Only two patients had transformed migraine. |
| Goadsby PJ. Study of occipital nerve stimulation for drug refractory migraine (PRISM UK). ClinicalTrials.gov 2011. Accessed 2 July 2012. Available from: <http://clinicaltrials.gov/show/NCT00747812> | ‘Drug refractory migraine’ – diagnosed criteria unclear. Study terminated - sponsor closed enrollment early based on interim data from the PRISM US Pivotal Study. |
| Hayek SM, Jasper JF, Deer TR, Narouze SN. Occipital neurostimulation-induced muscle spasms: implications for lead placement.[Erratum appears in Pain Physician. 2009 Nov-Dec;12(6):1027]. Pain Physician. 2009; 12(5): 867-76. | Technical notes. Case reports of five patients. |
| Jasper JF, Hayek SM. Implanted occipital nerve stimulators. Pain Physician. 2008; 11(2): 187-200. | Systematic review covering ONS for various headache disorders. Superseded by current review. |
| Jones RL. Occipital nerve stimulation using a medtronic resume II electrode array. Pain Physician. 2003; 6(4): 507-8. | Technical notes. |
| Kinfe TM, Schuss P, Vatter H. Occipital nerve block prior to occipital nerve stimulation for refractory chronic migraine and chronic cluster headache: Myth or prediction? Cephalalgia. 2014; DOI: 10.1177/0333102414541685 | Review focusing on the predictive value of occipital nerve block. |
| Lanteri-Minet M. French database of occipital nerves stimulation in the treatment of refractory chronic headache disorders (NGO). ClinicalTrials.gov 2013. Accessed 6 October 2014. Available from: http://clinicaltrials.gov/show/NCT01842763 | Ongoing registry – estimated completion December 2016. |
| Lipton, R. Occipital nerve stimulation (ONS) for migraine: OPTIMISE. ClinicalTrials.gov 2013. Accessed 9 August 2013. Available from: http://clinicaltrials.gov/ct2/show/NCT01775735 | Ongoing trial – estimated study completion June 2016 |
| Matharu MS, Bartsch T, Ward N, Frackowiak RS, Weiner R, Goadsby PJ. Central neuromodulation in chronic migraine patients with suboccipital stimulators: a PET study. Brain. 2004; 127(Pt:1): 1-30. | Case series of eight patients. Positron emission tomography study. |
| Paemeleire K, Van Buyten JP, Van BM, Alicino D, Van MG, Smet I, et al. Phenotype of patients responsive to occipital nerve stimulation for refractory head pain. Cephalalgia. 2010; 30(6): 662-73. | Mixed type of headache. Only included 8 patients with migraine. |
| Schwedt TJ, Dodick DW, Hentz J, Trentman TL, Zimmerman RS. Occipital nerve stimulation for chronic headache--long-term safety and efficacy. Cephalalgia. 2007; 27(2): 153-7. | Case series. Eight of the included patients had chronic migraine. |
| Shin JH, Kim YC, Jang IK, Kim JH, Park SY, Lee SC, et al. Occipital nerve stimulation in a patient with an intractable chronic headache -A case report. Korean Journal of Anesthesiology. 2011; 60(4): 298-301. | Single case report. |
| Trentman TL, Dodick DW, Zimmerman RS, Birch BD. Percutaneous occipital stimulator lead tip erosion: report of 2 cases. Pain Physician. 2008; 11(2): 253-6. | Case reports of two patients. |
| Trentman TL, Rosenfeld DM, Vargas BB, Schwedt TJ, Zimmerman RS, Dodick DW. Greater occipital nerve stimulation via the Bion microstimulator: implantation technique and stimulation parameters. Clinical trial: NCT00205894. Pain Physician. 2009; 12(3): 621-8. | Case series of nine patients. |
| Verrills P, Rose R, Mitchell B, Vivian D, Barnard A. Peripheral nerve stimulation for chronic headache: 60 cases and long-term follow-up. Neuromodulation. 2014; 14: 54-59 | Does not report separately on migraine patients |
| Vesper J, Slotty PJ, Bara G, Maciaczyk J, Gendolla A, Schu S. Occipital nerve stimulation for primary chronic headache disorders - A randomized trial on treatment efficacy (Abstract WIP-0247). Pain Practice. 2014; 14(Suppl1): 73. | Conference abstract for the crossover trial by Slotty and colleagues – reports results with mixed patients of chronic migraine (n=8), cluster headache (n=2) and tension headache (n=2). |

Appendix D: Characteristics of included case series

| **Study, location, centre & sample size** | **Follow-up & data collection** | **Diagnostic criteria** | **Other inclusion criteria/ type of pain** | **Treatment history** | **Trial stimulation and/or nerve block** | **Comments** |
| --- | --- | --- | --- | --- | --- | --- |
| Popeney & Aló, 2003 [[7](#_ENREF_7)] USA (Texas), single centre, n=25 | 18 months (mean, range 9 to 36); retrospective chart review & telephone interview. | ICHD-1 criteria for episodic migraine and suggested criteria for transformed migraine (Silberstein et al. 1994) | Headache ≥15 days per month and long-duration (≥ 4 hours) and were nonparoxysmal; progressive pain into the posterior occipital, vertex or retro-orbital regions. | Refractory to preventive and abortive medications (average 7); 76% medication overuse. | All patients responded to temporary bilateral occipital nerve blockade and all completed a successful 5-7 day trial stimulation. | None. |
|  |  |  |  |  |  |  |
| Oh et al. 2004 [[8](#_ENREF_8)] USA (Pittsburgh and Houston), two centres, n=10 | At 1 month and 6 months and as needed thereafter; office visit or telephone interview. | ICHD-1 criteria for episodic migraine and suggested criteria for transformed migraine (Silberstein et al. 1994) | Required pain intensity and duration not reported; ‘progressive cervical/skull base tension’ with secondary radiating posterior occipital, vertex, or retro orbital migrainous symptoms; | Failed at least 3 modes of conservative treatment (medication, physical therapy, blockade); medication overuse unclear. | ≥70% relief of pain with occipital anesthetic field block; all patients obtained immediate paresthesia and pain relief of >50% during ‘on the table’ trial | Total n=20 for the whole case series (including 10 other patients with occipital neuralgia). |
|  |  |  |  |  |  |  |
| Brewer et al. 2013 [[9](#_ENREF_9)] USA (Arizona), single centre, n=12 for migraine | Varied (1 to 70 months); retrospective chart review and telephone interview. | ICHD-1 and ICHD-2 | No specified inclusion criteria; unclear for other information. | Refractory to ≥7 preventive medications; medication overuse unclear. | ≥ 50% reduction in pain intensity or headache frequency during a 3-7 day trial stimulation; | Total n=29 for the whole case series with various types of headache disorders; patients who participated in industry trials were excluded |
|  |  |  |  |  |  |  |
| Kiss & Becker, 2012 [[10](#_ENREF_10)] Canada, single centre, n=10 | 33 months (median; range 7 to 38); prospective follow-up appointments. | ICHD-2 (chronic migraine) | Peak pain intensity ≥5 on a 0-10 scale; pain located between C3 level to vertex and any location between ears (same as ONSTIM). | Refractory to ≥ 2 acute and ≥2 prophylactic medications; medication overuse excluded (same as ONSTIM). | Successful temporary nerve block (≥50% reduction in pain) required. | All 10 patients were enrolled in the ONSTIM study described above. |
|  |  |  |  |  |  |  |
| Mammis et al. 2012 [[11](#_ENREF_11)] USA, single centre, n=24 for migraine | Varied (3 to 65 months); retrospective chart review. | Diagnoses were retrospectively classified according to ICHD-2 | No specific inclusion criteria; unclear for other information. | Treatment history not reported. | Successful trial stimulation of 4 to 7 days (significant reduction in the frequency and severity of the headache) was required | Total n=99 for the whole case series with various types of headache and craniofacial pain. Eight of the 24 migraine patients were enrolled in the RCT Silberstein et al. 2012 described above. |
|  |  |  |  |  |  |  |
| Palmisani et al. 2013 [[12](#_ENREF_12)] UK and Italy, two centres, n=17 for migraine | Varied (11 to 79 months); retrospective chart review and telephone interviews. | ICHD-2 | No specific inclusion criteria; candidates advised not to proceed where psychological evaluation indicated conditions that could be aggravated by treatment. | Failed to improve after 4 classes of preventative medicines and 3 classes of acute drugs with established efficacy. | ≥ 50% reduction in headache intensity and/or frequency associated with a decrease in headache medication use during a 7-10 day trial stimulation | Total n= 25 for whole case series including idiopathic intracranial hypertension, occipital neuralgia, cluster headaches and cervicogenic headache; 23 received implants. |
| Notaro et al. 2014 [[13](#_ENREF_13)] Italy, single centre, n=17 | 3 and 12 months; follow-up assessments and psychological interviews. | ICHD-2 | Met ICHD-2 criteria for chronic migraine; failure of previous drug therapies; MIDAS score >21; numeric rating scale score >5; no psychiatric condition; high compliance | ‘Failure of previous drug therapies’. | Mean improvement in MIDAS score 42% (±9.7) at 3 months and 46% ±28.2) at 12 months; Migration occurred in 5 patients; 3 IPGs removed after 8 months; 2 ex-plants |  |
|  |  |  |  |  |  |  |

ICHD – The International Classification of Headache Disorder, MIDAS - Migraine Disability Assessment

Appendix E: Stimulation devices and parameters used in included studies

|  | **Randomized controlled trials** | | | | | **Case series** | | | | |  |  |
| --- | --- | --- | --- | --- | --- | --- | --- | --- | --- | --- | --- | --- |
|  | **Lipton et al. 2009 (PRISM) [**[**1**](#_ENREF_1)**]** | **Saper et al. 2011 (ONSTIM) [**[**2**](#_ENREF_2)**]** | **Silberstein et al. 2012 [**[**3**](#_ENREF_3)**,** [**4**](#_ENREF_4)**]** | **Serra & Marchioretto 2012 [**[**5**](#_ENREF_5)**]** | **Slotty et al. 2014 [**[**6**](#_ENREF_6)**]** | **Popeney & Aló, 2003 [**[**7**](#_ENREF_7)**]** | **Oh et al. 2004 [**[**8**](#_ENREF_8)**]** | **Brewer et al. 2013 [**[**9**](#_ENREF_9)**]** | **Kiss & Becker, 2012 [**[**10**](#_ENREF_10)**]** | **Mammis et al. 2012 [**[**11**](#_ENREF_11)**]** | **Palmisani et al. 2013 [**[**12**](#_ENREF_12)**]** | **Notaro et al. 2014 [**[**13**](#_ENREF_13)**]** |
| **Lead type (model)** | Not stated | Cylindrical (Pisces Quad & Pisces Quad-Compact leads) | Cylindrical (Quattrode) | Cylindrical (quadripolar lead) | Cylindrical (Octrode^TM^) | Cylindrical (Pisces Quad Plus) | Cylindrical (not stated); Paddle (Resume TL) | Not stated | Cylindrical (Pisces Quad) | Cylindrical (not stated) | Cylindrical (not stated) | Cylindrical (Octad) |
| **Stimulation system** | Precision^TM^ | Synergy^TM^ & Synergy Versitrel^TM^ | Genesis^TM^ | Synergy Versitrel^TM^ | Not stated | Synergy^TM^ | Synergy^TM^ | Not stated | Synergy^TM^ | Not stated | Not stated | Genesis^TM^ |
| **Manufacturer** | Boston Scientific | Medtronic | St. Jude Medical | Medtronic | St. Jude Medical | Medtronic | Medtronic | Not stated | Medtronic | Not stated | Not stated | St. Jude Neuro modulation |
| **Stimulation parameters** | Active: 250 μsec pluses, 60 Hz, 0 to 12·7 mA Sham: 10 μsec pluses, 2 Hz, <1 mA,1 sec on 90 min off duty cycle | 60 to 450 µsec pulses, 3 to 130 Hz, 0 to 10·5 V | Not stated | Active: 330 to 450 μsec pluses, 50 Hz, ≤ 10·5 V Control: Off | Mean pulse width 335.8 µs (100-450, SD 120.7); mean frequency 37.9 Hz (30-80, SD 16.2); mean voltage 2.6 V (0.4-6.7, SD 1.8) in effective stimulation and 1.3V (0.3-3.1, SD 0.8) in subthreshould stimulation | Not stated | Not stated | Not stated | Not stated | Not stated | Not stated | ‘Determined according to the patient report’ and can be adjusted by the patients; details were not stated |

Appendix F: Results of additional outcomes

*Reduction in pain intensity*

In the ONSTIM study, a greater reduction in overall intensity (0-10 scale) was observed in the ONS group (1.5 ± 1.6) compared with sham control (0.5 ± 1.3) and medical management (0.6 ± 1.0) at 3-month follow-up. The difference between the ONS group and the two control groups were statistically significant ([calculated by the review author] ONS vs. sham, mean difference 1.00, 95%CI 0.13 to 1.87, p=0.002; ONS vs. medical management, mean difference 0.90, 95% CI 0.14 to 1.66, p=0.02). Silberstein and colleagues did not report mean reduction in pain intensity but they presented a ‘continuous proportion responder analysis’ based on pain intensity measured on VAS as described in the main text of this review. Serra and Marchioretto (2012) reported a reduced severity of headache measured on the Numerical Rating Scale in the ONS ‘on’ group compared with the ONS ‘off’ group (median 5 vs. 7.5, p<0.001 for the first period before crossover). Slotty et al reported a mean pain score on visual analogue scale (VAS) of mean visual analogue scale (VAS) score of 8.45 ± 0.99, 5.65 ± 2.11 and 1.98 ± 1.56 for no stimulation, subthreshold stimulation and suprathreshold stimulation respectively after one week of treatment.[[6](#_ENREF_6)] Reduction in pain intensity was not reported in the abstract for the study by Lipton et al.

*Quality of life*

Saper and colleagues reported significant improvement in SF-36 Mental Health domain for ONS group compared to medication management: 5.5 (SD 9.7) vs. -1.5 (SD 6.3), but the results for other domains and for the sham control group were not reported [[2](#_ENREF_2)]. In the trial by Silberstein and colleagues, 66.7% of patients treated with ONS and 17.2% treated with sham control reported improved quality of life at three months [[14](#_ENREF_14)]. Improved quality of life was reported in 58% (91/157) of all randomized patients at one year [[4](#_ENREF_4)].

Similar and significant improvements from baseline in mental component summary (from 35.9 ± 8.2 to 43.3 ± 5.8 at 12 months) and physical component summary scores (from 42.9 ± 5.8 to 45.4 ± 4.8) were observed throughout the follow-up between 1 to 12 months in the whole cohort (uncontrolled) of the crossover trial by Serra and Marchioretto [[5](#_ENREF_5)]. Slotty et al. reported no significant difference in the mean SF-36 score between effective (suprathreshold) stimulation (64.75 ± 12.79), subthreshold stimulation (66.50 ± 13.34) and no stimulation (67.38 ± 12.34) at the end of one week period in their crossover RCT [[6](#_ENREF_6)].

*Satisfaction with treatment*

Sixty-six percent of patients in the ONS group reported satisfaction with treatment compared to 25% in the medication management group in the ONSTIM study (results for the sham control group were not reported) [[2](#_ENREF_2)]. Fifty-one percent of patients in the ONS groups compared to 19% in the sham control group were satisfied with treatment in the RCT reported by Silberstein and colleagues at three months[[14](#_ENREF_14)]. 66% (104/157) of all randomized patients were satisfied or very satisfied with treatment at one year [[4](#_ENREF_4)].

*Physical and mental functions*

Saper and colleagues reported a significantly greater reduction in the Profile of Moods States (POMS) score for ONS group compared to sham control and medication management groups (8.7 [SD 12.0] vs. 1.6 [SD 10.1] and 0.4 [SD 9.4] respectively) and greater improvement in functional disability scale for the ONS group compared to the medication management group (0.3 [SD 0.5] vs. 0.0 [SD 0.3])[[2](#_ENREF_2)]. Silberstein and colleagues reported greater improvement in the Zung Pain and Distress Scale score for ONS group compared to sham control (13.3 vs. 5.5, SD not reported, p<0.01) at three months [[14](#_ENREF_14)] and a significant improvement from baseline in total score and pain, mood and behaviour subcomponent score at one year (results from ONS and control groups were combined).[[4](#_ENREF_4)]

*Serious adverse events*

Three patients (3/56, 6%) experienced serious adverse events requiring hospitalization in the ONSTIM study [[2](#_ENREF_2)]. These were related to implant site infection, lead migration and post-operative nausea. Silberstein and colleagues reported in their conference abstract two cases of serious device- or procedure-related events (2/157, 1%) at 3 months, including one case of infection and one case of post-operative pain that required hospitalization [[14](#_ENREF_14)]. Forty serious adverse events were recorded at one year as described in the main text[[4](#_ENREF_4)] Serra and Marchioretto stated that there were two ‘severe’ (not defined) implantation site infections and two severe lead dislocations in their case series (4/30, 13%)[[5](#_ENREF_5)], whereas Kiss and Becker reported two cases of psychiatric complications requiring hospitalizations (2/10, 20%) [[10](#_ENREF_10)]. One of the patients with pre-existing stable bipolar disorder required a 3-week hospital stay for narcotic addiction 10 months post implant; the other patient with no prior psychiatric issues experienced significant depression requiring inpatient management. It was not clear whether any causality assessment was carried out for these cases. In the trial terminated early by its sponsor [[15](#_ENREF_15)], serious adverse events were reported in six out of nine (67%) patients enrolled. Events listed include umbilical hernia, therapeutic product ineffective, implant site pain, device ineffective, joint range of motion decreased, intervertebral disc protrusion, headache and proteinuria. Serious adverse events either did not occur or were not reported in other studies.

Appendix G: Long-term effectiveness results from uncontrolled phases of RCTs and case series

|  | **RCTs** |  | **Case series** |  |  |  |  |  |
| --- | --- | --- | --- | --- | --- | --- | --- | --- |
| **Study** | **Serra & Marchioretto 2012 [**[**5**](#_ENREF_5)**]** | **Silberstein et al. 2012 (Dodick et al. 2014) [**[**4**](#_ENREF_4)**]** | **Propeney & Aló 2003 [**[**7**](#_ENREF_7)**]** | **Kiss & Becker 2012 [**[**10**](#_ENREF_10)**]** | **Mammis et al. 2012 [**[**11**](#_ENREF_11)**]** | **Brewer et al. 2013 [**[**9**](#_ENREF_9)**]** | **Palmisani et al. 2013 [**[**12**](#_ENREF_12)**]** | **Notaro et al. 2014 [**[**13**](#_ENREF_13)**]** |
| **Sample size** | 30 | 157 | 25 | 10 | 21 | 12 | 17 | 17 |
|  |  |  |  |  |  |  |  |  |
| **Follow-up** | 1 year | 1 year | Mean 18 months (range 9 to 36) | Median 33 months (range 7 to 38) | Range 3 to 65 months | Range 1 to 70 months | Mean 40 ± 27 months (range 11 to 77) | 1 year |
|  |  |  |  |  |  |  |  |  |
| **Long-term use** | 97% (29/30) continued ONS at 1 year | 88% (133/151*) continued ONS at 1 year | 100% (25/25) continued ONS at last follow-up | 50% (5/10) continued ONS at last follow-up | 90% (19/21) continued ONS at last follow-up | 58% (7/12) continued ONS for between 9 and 70 months | 59% (10/17) continued ONS at last follow-up | 82% (14/17) continued ONS at 1 year |
|  |  |  |  |  |  |  |  |  |
| **Headache days and frequency** | Not reported | Days with prolonged, moderate or severe headache N=111 (71%) Baseline: 21.6 ± 7.1 Reduction at 1 year: 6.7 ± 8.4 (p<0.001) | Headache frequency per 90 days N=25 (100%) Baseline: 75.56 ± 26.81; Post-stimulation: 37.45 ±7.49 (p<0.001) | Not reported | Not reported | Result for chronic migraine was not reported separately | Not reported | Not reported |
|  |  |  |  |  |  |  |  |  |
| **MIDAS** | N=29 (97%) Total score, median (IQR) Baseline: 79 (30-135) 1 year: 10 (0-20) p<0.001; MIDAS – A, median (IQR) Baseline: 70 (50-88); 1 year: 14 (8-16) (p<0.001); MIDAS – B, median (IQR) Baseline: 8 (7-8); 1 year: 5 (4-6) (p<0.001) | N=142 (90%, baseline) and 133 (85%, 1 year) Baseline: 156.6 ± 75.3 Reduction at 1 year: 50.9 ±71.9 (p<0.001) | N=25 (100%) Baseline: 121 ± 56 (range 29-240); Post-stimulation: 15 ± 25.1 (range 0-90); Reduction: 88.7 ± 1.72 (range 500-100) | Not reported | Not reported | Result for chronic migraine was not reported separately | Not reported | N=17 (100%) Baseline: 45.3 ± 13.8; 1 year: 22.1 ± 9.74; Improvement: 46% ± 28.2% (p<0.05) |
|  |  |  |  |  |  |  |  |  |
| **Responder rate: reduction in headache days and/or pain intensity (VAS) and/or frequency** | Not reported | N=111 (71%) at 1 year:30% reduction: 59.5%; 50% reduction: 47.8% | N=25 (100%) ≥50% reduction: 88% | Not reported | Not reported | N=12 (100%) Successful treatment judged by the investigator: 42% | N=17 (100%) 50% reduction: 53% | Not reported |
|  |  |  |  |  |  |  |  |  |
| **Pain / headache severity** | Not reported | Zung PAD scale. Total score: N=132 (84%); Baseline: 66.8 ± 13.6 Reduction at 1 year: 10.3 ± 14.8 (p<0.001). Pain subcomponent score: N=131 (83%), Baseline 78.8 ± 25.7; Reduction at 1 year: 9.0 ± 27.0 (p<0.001). Patient reported pain relief at 1 year: 49.5% ± 30.7% | Headache severity (0-10), N=25 (100%) Baseline: 9.32 ± 1.28; Post-stimulation: 5.72 ± 3.31 9p<0.001) | Not reported | Not reported | Result for chronic migraine was not reported separately | Not reported | Numerical rating scale, N=17 (100%, 1 year); Baseline: 9.8 ± 0.7; 1 year: 5.7 ± 2.6 (p<0.05); Improvement: 40% ± 30.5% |
|  |  |  |  |  |  |  |  |  |
| **Quality of life** | SF-36, N=29 (97%), *Physical Component Summary*, Baseline: 42.9 ± 5.8; 1 year: 45.4 ± 4.8. *Mental Component summary* Baseline: 35.9 ± 8.2; 1 year: 43.3 ± 5.8 (p<0.05) | Patient reported quality of life (improved, stayed the same, deteriorated), N=133 (85%); Improved: 68.4% | Not reported | Not reported | Not reported | Not reported | Not reported | SF-36, N=17 (100%); *Physical Component Summary*, Baseline: 29.8 ± 3; 1 year: 40.3 ± 7 (p<0.05). *Mental Component Summary,* Baseline: 26.5 ± 6.6; 1 year: 52.3 ± 6.7 (p<0.05) |
| **Other outcomes** | Drug intake, median monthly dose. Triptans, N=22 (73%), Baseline: 20; 1year: 3. NSAIDs, N=16 (53%), Baseline 25.5; 1 year 2 (p<0.001) | Zung PAD scale, Mood subcomponent score, N=132 (84%); Reduction at 1year: 12.9 (SD not reported) (p<0.001). Zung PAD scale, Behaviour subcomponent score, N=132 (84%), Reduction at 1 year: 9.2 (SD not reported) (p<0.001). Patient reported headache relief (excellent, good, fair, poor), N=133 (85%): Excellent or good at 1 year: 65.4%. Satisfied with device, N=133 (85%): 58.6%; Satisfied or very satisfied with the results of procedure, N=153 (97%): 68%; Willing to undergo same procedure again, N=141 (90%): 83%; Would recommend the procedure to someone else, N=151 (96%): 88.7% | Disability, N=25 (100%). Baseline: 100% severe disability. Post-stimulation: 20% severe, 16% moderate, 4% mild, 60% little or no disability | Not reported | Not reported | Not reported | Not reported | Not reported |

Results shown are mean ± standard deviation unless otherwise specified. The percentages shown in brackets next to N (number included in analysis) are the proportion of patients who contributed to the long-term data out of the original sample sizes.

*Six patients lost to follow-up or dropped out for other reasons

IQR: inter-quartile range; MIDAS: Migraine Disability Assessment; NSAIDs: non-steroidal anti-inflammatory drugs; PAD: Pain and Distress; VAS: visual analogue scale.

Appendix H: GRADE summary of findings table

| **Outcomes**  **(effectiveness)** | **Illustrative, comparative mean or risks** | | | **Number of participants (studies)** | **Quality of the evidence (GRADE)** | **Comments** |
| --- | --- | --- | --- | --- | --- | --- |
|  |  |  |  |  |  |  |
| Days with prolonged (≥4 hr) moderate or severe headache per months | Assumed mean: at baseline: 20 days;- with sham stimulation: 17 days | Corresponding mean with ONS: 14.4 days | Intervention effect: reduction of 2.6 (95% CI 0.9 to 4.3) days per month compared with sham stimulation | 326 (3 studies) | Moderate (due to risk of bias) | Based on RCT follow-up at 3 months |
|  |  |  |  |  |  |  |
| Responder rates (achieving ≥50% improvement*) | Assumed responder rate for sham stimulation: range from 6 to 13 per 100 patients | Corresponding responder rate with ONS: 12% to 26% | More likely to become a responder with ONS, relative risk 2.07 (0.50 to 8.55) | 207 (2 studies) | Low (due to risk of bias, imprecision and inconsistency) | Based on RCT follow-up at 3 months; very wide confidence interval crossing the line of no effect |
|  |  |  |  |  |  |  |
| **Outcomes (adverse events)** | **Observed event rates** | | | **Number of participants (studies)** | **Quality of the evidence (GRADE)** | **Comments** |
|  |  |  |  |  |  |  |
| Lead migration | Range from 13 to 24 per 100 patients at 3 months; and from 10 to 18 per 100 patients at 1 year | | | 238 (3 studies) | Low (due to risk of reporting bias, imprecision and inconsistency) | Based on data from RCTs (see Figure 3A); higher rates have been reported in case series of varied duration of follow-up |
|  |  |  |  |  |  |  |
| Infection | Range from 4 to 14 per 100 patients at 3 months; and 7 per 100 patients at 1 year | | | 238 (3 studies) | Low (due to risk of reporting bias and imprecision) | Based on data from RCTs (see Figure 3B); higher rates have been reported in case series of varied duration of follow-up |

RCT: randomized controlled trial. *Definitions varied between studies – see Appendix 2.

**References for Appendices**

1. Lipton RB, Goadsby PJ, Cady RK, Aurora SK, Grosberg BM, et al. (2009) PRISM study: occipital nerve stimulation for treatment-refractory migraine [abstract PO47]. Cephalalgia 29: 30.
2. Saper JR, Dodick DW, Silberstein SD, McCarville S, Sun M, et al. (2011) Occipital nerve stimulation for the treatment of intractable chronic migraine headache: ONSTIM feasibility study. Cephalalgia 31: 271-285.
3. Silberstein SD, Dodick DW, Saper J, Huh B, Slavin KV, et al. (2012) Safety and efficacy of peripheral nerve stimulation of the occipital nerves for the management of chronic migraine: Results from a randomized, multicenter, double-blinded, controled study. Cephalalgia 32: 1165-1179.
4. Dodick DW, Silberstein SD, Reed KL, Deer TR, Slavin KV, et al. (2014) Safety and efficacy of peripheral nerve stimulation of the occipital nerves for the management of chronic migraine: Long-term results from a randomized, multicenter, double-blinded, controlled study. Cephalalgia. Published online first. DOI: 10.1177/0333102414543331
5. Serra G, Marchioretto F (2012) Occipital nerve stimulation for chronic migraine: A randomized trial. Pain Physician 15: 245-253.
6. Slotty P, Bara G, Kowatz L, Gendolla A, Wille C, et al. (2014) Occipital nerve stimulation for chronic migraine: A randomized trial on subthreshold stimulation. Cephalalgia. Published online first. DOI: 10.1177/0333102414534082.
7. Popeney CA, Alo KM (2003) Peripheral neurostimulation for the treatment of chronic, disabling transformed migraine. Headache 43: 369-375.
8. Oh MY, Ortega J, Bellotte JB, Whiting DM, Alo K (2004) Peripheral nerve stimulation for the treatment of occipital neuralgia and transformed migraine using a c1-2-3 subcutaneous paddle style electrode: a technical report. Neuromodulation 7: 103-112.
9. Brewer AC, Trentman TL, Ivancic MG, Vargas BB, Rebecca AM, et al. (2013) Long-term outcome in occipital nerve stimulation patients with medically intractable primary headache disorders. Neuromodulation 16: 557-562.
10. Kiss ZHT, Becker WJ (2012) Occipital stimulation for chronic migraine: Patient selection and complications. Canadian Journal of Neurological Sciences 39: 807-812.
11. Mammis A, Sinclair 3rd GL, Mogilner AY (2012) Peripheral neuromodulation for headache and craniofacial pain: indications, outcomes, and complications from a single center. Clinical Neurosurgery 59: 114-118.
12. Palmisani S, Al-Kaisy A, Arcioni R, Smith T, Negro A, et al. (2013) A six year retrospective review of occipital nerve stimulation practice--controversies and challenges of an emerging technique for treating refractory headache syndromes. The Journal of Headache and Pain 14: 67.
13. Notaro P, Buratti E, Meroni A, Montagna MC, Rubino FG, et al. (2014) The effects of peripheral occipital nerve stimulation for the treatment of patients suffering from chronic migraine: a single center experience. Pain Physician 17: E369-374.
14. Silberstein S, Dodick D, Saper J, Huh B, Reed K, et al. (2011) The safety and efficacy of occipital nerve stimulation for the management of chronic migraine [abstract]. Cephalalgia 31: 117.
15. Goadsby PJ (2011) Study of occipital nerve stimulation for drug refractory migraine (PRISM UK). URL: <http://clinicaltrials.gov/show/NCT00747812>. Last updated 28 June 2012. .
